# Evaluation of the biological efficiency of *Terminalia chebula* fruit extract against neurochemical changes induced in brain of diabetic rats: an epigenetic study

**DOI:** 10.1007/s10787-024-01428-9

**Published:** 2024-02-08

**Authors:** Marwa E. A. El-Shamarka, Wael Mahmoud Aboulthana, Nagwa Ibrahim Omar, Marwa M. Mahfouz

**Affiliations:** 1 Department of Narcotics, Ergogenic Aids and Poisons, Medical Research Institute, National Research Centre, 33 El Bohouth St. (Former El Tahrir St.), P.O. 12622, Dokki, Giza, Egypt; 2 Biochemistry Department, Biotechnology Research Institute, National Research Centre, 33 El Bohouth St. (Former El Tahrir St.), P.O. 12622, Dokki, Giza, Egypt; 3 Department of Pharmacology and Toxicology, Faculty of Pharmacy, Menoufia University, Shibīn Al-Kawm, Egypt

**Keywords:** Diabetes mellitus, Neurodegeneration, Oxidative stress, *Terminalia chebula*, Electrophoretic isoenzymes

## Abstract

Diabetes mellitus (DM) is a chronic and progressive metabolic disorder that can stimulate neuroinflammation and increase oxidative stress in the brain. Therefore, the present study was aimed to assess the efficacy of ethanolic* Terminalia chebula* extract against the neurochemical and histopathological changes induced in the brains of diabetic rats. The study clarified the reduction in oxidative stress induced in the brains of diabetic rats by the significant (*P* ≤ 0.05) increase in levels of the antioxidants with decreasing the peroxidation products via ethanolic *T. chebula* extract at both doses (400 and 600 mg/kg). Moreover, *T. chebula* extract improved the brain integrity by lowering levels of interleukin-1β (IL-1β), tumor necrosis factor-α (TNF-α), β-amyloid (Aβ) content, monocyte chemoattractant protein-1 (MCP-1) and acetylcholine esterase (ACHE) significantly (*P* ≤ 0.05) in a dose dependent manner compared to brain of diabetic rats. Severe nuclear pyknosis and degeneration were noticed in neurons of the cerebral cortex, hippocampus and striatum in brains of diabetic rats. The severity of these alterations decreased with *T. chebula* extract at a dose of 600 mg/kg compared to the other treated groups. The different electrophoretic protein and isoenzyme assays revealed that the lowest similarity index (SI%) values exist in the brains of diabetic rats compared to the control group. The quantity of the most native proteins and isoenzyme types increased significantly (*P* ≤ 0.05) in the brains of diabetic rats, and these electrophoretic variations were completely diminished by *T. chebula* extract. The study concluded that *T. chebula* extract ameliorated the biochemical, histopathological and electrophoretic abnormalities induced in the brains of diabetic rats when administered at a dose of 600 mg/kg.

## Introduction

Diabetes mellitus (DM) is categorized as one of the chronic metabolic disorders that is characterized by hyperglycemia with subsequent insulin resistance and is considered a prominent cause of death worldwide. According to the survey carried out by the International Diabetes Federation (IDF) during 2017, it was expected that the rate of disease incidence would increase more and more in the next few years (Søfteland et al. 2019).

The DM induces several complications, not only in peripheral organs but also in the central nervous system, due to alterations in the glycometabolism (Stranahan 2015). Initially, the effects of diabetes on the brain may be undetectable, but the gradual decrease in blood supply to neurons can finally cause brain atrophy (Yarube and Mukhtar 2018). Diabetes has an increased risk of developing neurological complications such cognitive impairment (brain fog), vascular dementia, diabetic neuropathy, Alzheimer’s disease, and other neurodegenerative diseases (Nduohosewo and Ekong 2020). The DM is commonly associated with several neuropsychiatric comorbidities, such as depression, schizophrenia, and bipolar disorder (Ringin et al. 2023). The Hippocampal dysfunction, such as memory dysfunction, is considered one such complication that is associated with disability and the development of dementia, Alzheimer’s disease, and depression (Weerasinghe-Mudiyanselage et al. 2022). Diabetic encephalopathy, diabetes-associated cognitive decline, cerebral impairment and central neuropathy have been used to describe mild to moderate diabetes-related cognitive dysfunction (Li et al. 2019).

The changes induced by oxidative stress in the structure and function of the macromolecules (proteins, lipids and DNA) are related to the etiology of diabetes and hypothalamic–pituitary–adrenal axis dysregulation that may be attributed to impaired neurogenesis and the synthesis of brain-derived neurotrophic factor (Zanoveli et al. 2016). Therefore, it is necessary to understand the etiology of the disease to innovate therapeutic strategies for hippocampal memory dysfunction induced by diabetes.

Metformin is considered one of the first-line anti-diabetic drugs commonly used for treating DM (Zhou et al. 2018). It exhibits its hypoglycemic activity by decreasing the production of hepatic glucose and increasing the utilization of glucose by skeletal myocytes (Turban et al. 2012). Therefore, the experimental studies carried out on animal models revealed the efficiency of alternative anti-diabetic agents compared to those of metformin, which is a commercially available drug. In 2021, Kułaczkowska et al. (2021) postulated that it is necessary to re-evaluate the efficacy and therapeutic effect of metformin due to the multifactorial mechanisms of the disease and its complications. Due to the serious micro- and macro-vascular complications of the disease, no single medication is absolutely effective for the treatment of the disease. Therefore, it is necessary to undergo further studies to search for new medications that aid in attenuating the progression of the disease and its possible complications (Izzo et al. 2021). Kashtoh and Baek (2023) proposed that plant derived drugs with anti-diabetic properties are frequently considered to be cheaper and have low toxicity compared to the other synthetic ones.

*Terminalia chebula* (*T. chebula*) is native to Southeast Asia and India. It is mentioned as the "King of Medicines" in Ayurvedic Materia Medica and used in Egyptian folk medicine. It is well known that *T. chebula* fruits are rich in various active phyto-constituents like tannins, polyphenols and triterpenoids that are categorized as powerful antioxidant, antifungal, anti-inflammatory, anti-cancer, anti-mutagenic and anti-diabetic agents in addition to their maltase inhibitory activity (Sheng et al. 2018). Choi et al. (2015) demonstrated that the *T. chebula* extract exhibited a protective effect against a hepatic injury model due to its antioxidant capacities and scavenging activity, in addition to modulating inflammatory reactions. A recent study carried out by Eltimamy et al. (2022) showed that the ethanolic* T. chebula* extract has anti-diabetic, anti-lipidemic, hepatoprotective and renoprotective effects against DM and this is probably attributed to the promotion of insulin release beside the insulin-like action of its phyto-constituents (Abu-Odeh and Talib 2021). Therefore, the present study was designed to appraise the efficiency of ethanolic* T. chebula* extract against neuroinflammation and oxidative stress induced in the brains of diabetic rats.

## Material and methods

### Preparation of *Terminalia chebula* fruit extract

*Terminalia chebula* fruits were collected from Indore, Madhya Pradesh, India, and dried in an incubator at 50 °C for 72 h. Consequently, the dried fruits were crushed and ground into powder (0.5 kg) by an electric blender, then percolated with ethanol (75%) for 7 days using the cold maceration process as suggested by Lwin et al. (2020). The peel particles were removed by filtering the mixture through *Whatman No.* 2 filter paper, and the filtrate obtained was concentrated at 40–50 °C using a rotary evaporator under reduced pressure to remove the solvent. The viscous extract obtained by drying the semisolid viscous mass in a water bath at 50 °C for 48 h exposed to air to be converted completely into dried powder. The residue (dry extract) was suspended in 2 mL of ethanol (96%) and the total volume increased to 100 mL in the volumetric flask by adding distilled water. The extract was daily injected orally at doses of 400 and 600 mg/kg (Abdel-Salam et al. 2014).

### Quantitative determination of major phytoconstituents

The concentration of total polyphenolic compounds in the ethanolic *T. chebula* fruits extract was determined as mg gallic acid/100 gm using the method described by Singleton and Rossi (1965). During this method, folin ciocalteu reagent (0.25 mL) was added to the diluted extract (1:5) followed by an aqueous sodium carbonate solution (1.25 mL). All tubes were vortexed then allowed to sit at room temperature for 40 min. Absorbance of blue-colored mixtures was recorded at 725 nm against a blank containing distilled water (0.5 mL) instead of extract. The concentration of total polyphenols was calculated from the calibration curve of various concentrations of gallic acid standard solutions. The total tannin content of the extract was assessed by the method proposed by Broadhurst and Jones (1978) using tannic acid as a reference compound. In brief, the extract (400 µL) was added to 3 mL of a solution of vanillin (4% in methanol) and 1.5 mL of concentrated hydrochloric acid. After 15 min of incubation, the absorbance was measured at 500 nm and the concentration was obtained from the calibration curve of various concentrations (20, 40, 50, 80 and 100 mg/L) of tannic acid.

### In vitro biological activities

Total antioxidant capacity (TAC) was assessed as mg gallic acid/gm of dry weight, based on the method documented by Prieto et al. (1999). Briefly, the extract (0.1 mL) was combined with 1 mL of reagent solution (0.3 N sulfuric acid, 28 mM sodium phosphate, and 4 mM ammonium molybdate). Methanol (80%) was used instead of the extract for the blank. The tubes were capped and incubated in a boiling water bath for 90 min. Then, the samples were cooled to room temperature, and the absorbance was measured at 695 nm against a blank.

Total iron reducing power (IRP) was assayed as µg/mL by the method suggested by Oyaizu (1986), using ascorbic acid as a standard. Briefly, the extract (1 mL) was mixed with 1 mL of sodium phosphate buffer (200 mM, pH 6.6) and 1 mL of potassium ferricyanide (1%). Subsequently, the mixture was incubated at 50 °C for 20 min, followed by adding 1 mL of trichloroacetic acid (10% w/v). The mixture was centrifuged at 2000 rpm for 10 min. The upper layer solution (2.5 mL) was mixed with 2.5 mL of double deionised water and 1 mL of fresh ferric chloride (0.1%). The absorbance was measured at 700 nm against blank prepared without adding extract. A high absorbance of the reaction mixture indicates a higher reducing power.

The scavenging activities of the extract were assayed against 1,1-Diphenyl-2-picryl-hydrazyl (DPPH) and 2,2′-azinobis-(3-ethylbenzothiazoline-6-sulfonic acid) (ABTS) radicals. The median inhibitory concentration (IC_50_) against DPPH radical was calculated according to the method suggested by Rahman et al. (2015), who demonstrated that the extract (1 mL) was added to a 1 mL DPPH radical solution in methanol (final DPPH concentration, 0.2 mM). The reaction mixture was vortexed and incubated at 37 °C under dim light for 30 min. The absorbance of the resulting solution was measured at 517 nm. Then, the inhibition percentage (%) was plotted against concentration, and from the graph, IC_50_ was calculated.

The percentage of inhibitory effect against ABTS was determined using ascorbic acid as a standard according to the method demonstrated by Arnao et al. (2001). The stock solutions were consisting of ABTS solution (7 mM) and potassium persulfate solution (2.4 mM). The working solution was then prepared by mixing the two stock solutions in equal quantities and allowing them to react for 14 h at room temperature in a dark place. The solution was then diluted by mixing 1 mL ABTS solution, with 60 mL methanol. The extracts (1 mL) were allowed to react with 1 mL of a freshly prepared ABTS solution and the absorbance was taken at 734 nm after 7 min using a spectrophotometer. The ABTS scavenging capacity of the extract was compared with that of ascorbic acid.

The anti-diabetic activity was assessed by calculating inhibition percents (%) of α-amylase and α-glucosidase enzymes according to the methods demonstrated by Wickramaratne et al. (2016) and Pistia-Brueggeman and Hollingsworth (2001), respectively, using acarbose as a positive control. In brief, 0.5 mL of extract was mixed with 0.5 mL of α-amylase solution (0.5 mg/mL) in buffer (Na_2_HPO_4_/NaH_2_PO_4_ (0.02 M), NaCl (0.006 M) at pH 6.9) to give concentrations ranging from 25 to 800 μg/mL. The mixture was incubated at room temperature for 10 min, and 200 μL of the starch solution (1% in water (w/v) buffer [Na_2_HPO_4_/NaH_2_PO_4_ (0.02 M), NaCl (0.006 M) at pH 6.9)] was added. The reaction was terminated by adding 200 μL 3.5-dinitrosalicylic acid (DNSA) (coloring) reagent (12 g of sodium potassium tartrate tetrahydrate in 8.0 mL of 2 M NaOH and 20 mL of 96 mM of DNSA solution). At this time, the test tubes were placed in a boiling water bath (100 °C) for 10 min, and the mixture was cooled to ambient temperature and diluted with 5 mL of distilled water. The absorbance was measured at 540 nm. Moreover, the activity of α-glucosidase enzyme was estimated by incubating the extract (50 μL) with 10 μL of the α-glucosidase enzyme solution (1 U/mL) for 20 min at 37 °C with an additional 125 μL of 0.1 M phosphate buffer (pH 6.8). After 20 min, the reaction was started with the addition of 20 μL of 1 M p-nitrophenyl-α-D-glucopyranoside (pNPG) (substrate), and the mixture was incubated for 30 min. The reaction was terminated with the addition of 50 μL of Na_2_CO_3_ (0.1 N), and final absorbance was measured at 405 nm.

During the assay of the anti-Alzheimer's activity, the inhibition percent (%) of the acetylcholinesterase (AChE) enzyme was assessed by Ellman's method (Ellman et al. 1961) using donepezil as the standard drug. The extract (5 µL dissolved in a phosphate buffer (0.1 M, pH 8)) was added to 5 µL of Acetylthiocholine (ATCh) (0.5 mM) and 5 µL of 5,5′-dithiobis-2-nitrobenzoic acid (DTNB) (0.03 mM), then mixed and incubated at 30 °C for 10 min. Then, 5 µL of AChE (0.3 U/mL) solution was added to the initial mixture to start the reaction, and then absorbance was determined at 412 nm.

The anti-inflammatory activity was measured by calculating the inhibition percent (%) of protein denaturation (Das and Sureshkumar 2016) and proteinase activity (Oyedapo and Famurewa 1995) using diclofenac sodium as the standard drug. The extract (0.5 mL) was mixed with 0.45 mL of bovine serum albumin (BSA) (5% w/v aqueous solution) and 0.05 mL distilled water. Consequently, the test solution (0.05 mL) was added to 0.45 mL of distilled water to form the product control (0.5 mL). The standard solution (0.5 mL) was prepared by mixing 0.45 mL of BSA with 0.05 mL of diclofenac sodium. The tested extract and diclofenac sodium (standard) were used. The pH value in all prepared solutions was adjusted to 6.3 using HCl (1 N). All the samples were incubated at 37 °C for 20 min, then the temperature increased to 57 °C and the samples were maintained at that degree for 3 min. Phosphate buffer (2.5 mL) was added to the prepared solutions after cooling. The absorbance was determined at 416 nm. The inhibition percent (%) of protein denaturation was calculated. The proteinase inhibitory activity was determined by adding the extract (1 mL) to a reaction mixture consisting of 0.06 mg trypsin dissolved in 1 mL of 20 mM Tris HCl buffer (pH 7.4). After incubating the mixture for 5 min at 37 °C, 1 mL of casein (0.8% w/v) was added. Then, it was incubated for an additional 20 min, followed by adding 2 mL perchloric acid (70%) to terminate the reaction. After centrifugation of the cloudy suspension, the absorbance of the supernatant was determined at 210 nm against buffer as the blank, and the inhibition percent (%) of proteinase activity was calculated.

### Animals and treatments

Healthy fifty adult male Wistar albino rats (weighing 170–200 g) were kept under normal environmental conditions and provided with standard food and water ad libitum.

#### Induction of diabetes mellitus

The streptozotocin (STZ) solution was freshly prepared in citrate buffer (100 mM, pH 4.5), then injected intraperitoneally (*i.p*.) to rats after fasting overnight at a dose of 60 mg/kg b.w. (Archana et al. 2001). Massive glycosuria and hyperglycemia occurred within a few days after injecting STZ solution. The concentration of fasting blood glucose was measured after 72 h of STZ injection to confirm the induction process. Rats were considered diabetic when their blood glucose level was higher than 200 mg/dl.

#### Experimental design

Fifty rats were divided randomly into five groups (ten per group). Control group: Rats received distilled water and were fed a normal diet ad libitum for 21 days. Diabetic group: Rats were injected with a single dose of STZ solution *i.p*., and the diabetic rats were sacrificed after 21 days. Diabetic rats treated with metformin: Rats were injected with STZ solution and treated after 72 h of STZ injection with metformin as a standard drug at a dose of 150 mg/kg to obtain euglycemia for 21 days. Diabetic rats treated with *T. chebula* extract (400 mg/kg): Rats were injected with STZ solution and treated with ethanolic* T. chebula* fruit extract at a dose of 400 mg/kg for 21 days. Diabetic rats treated with *T. chebula* extract (600 mg/kg): Rats were injected with STZ solution and treated with ethanolic* T. chebula* fruit extract at a dose of 600 mg/kg for 21 days.

### Collection of samples

Rats were anesthetized and sacrificed at the end of the experiment (i.e., on the 21st day) by cervical dislocation. The brain tissue was excised and washed in ice-cold saline. A few autopsied pieces were preserved in neutral buffered formalin solution (10%) for histopathological investigation, and the other pieces were homogenized by Tissue Master TM125 (Omni International, USA) in potassium phosphate buffer (pH 7.4) and centrifuged for 10 min at 3000 rpm. Aliquots of these homogenates were used for different biochemical and electrophoretic assays.

### Biochemical assays

The total antioxidant capacity (TAC) (Koracevic et al. 2001) and reduced glutathione (GSH) (Beutler et al. 1963) were quantified in brain tissue homogenates as µmol/g and mg/g tissue, respectively. Concentrations of lipid peroxidation product (LPO) (Ohkawa et al. 1979) and total protein carbonyl content (TPC) (Levine et al. 1994) were measured as nmol/g and nmol/mg Protein, respectively.

The quantitative sandwich enzyme immunoassay technique was used for quantifying levels of interleukin-1β (IL-1β), tumor necrosis factor-α (TNF-α), β-amyloid (Aβ) and Aβ Fragment 1–42 contents that were expressed in supernatants of brain tissue homogenates as Pg/g using enzyme-linked immunosorbent assay (ELISA) Kits (USCN Life Science, Inc.). The monocyte chemoattractant protein-1 (MCP-1) was assessed as Pg/g using a Rat MCP-1 solid-phase sandwich ELISA. Brain acetylcholineesterase (ACHE) activity was determined in tissue homogenates as ng/g using the Ellman Method, proposed by Ellman et al. (1961) and modified by Gorun et al. (1978).

### Histopathological examination

After sacrifice, the preserved brain specimens were dehydrated in serial concentrations of alcohol solutions and used for preparing paraffin sections with a thickness 5 μm, then stained with Hematoxylin and Eosin (H&E) for histopathological examination as proposed by Suvarna et al. (2019). The severity of the histopathological changes was scored as the mean of at least five rats, based on as method suggested by Dommels et al. (2007). For each investigated section, the scores were assigned between 0 (no damage) and +++ (maximal damage).

### Electrophoretic assays

#### Native electrophoretic patterns

The known weight of brain tissue was homogenized in extraction buffer and centrifuged. From each group, equal volumes of the individual supernatants were mixed in one tube and used as one sample. The concentration of total protein was quantified in all pooled samples, as demonstrated by Bradford (1976). All the samples were diluted with loading dye to make the protein concentrations equal in all wells during the electrophoretic assays.

The Polyacrylamide Gel Electrophoresis (PAGE) was used for separating the native proteins that were stained with commassie brilliant blue (CBB) (Darwesh et al. 2015) for assaying the protein bands that appeared as blue bands, stained with Sudan Black B (SBB) (Subramaniam and Chaubal 1990) for assaying the lipid moiety of native protein that appeared as black bands, and stained with Alizarin Red "S" (Abd Elhalim et al. 2017) for assaying the calcium moiety of native protein that appeared as yellow bands.

The electrophoretic catalase (CAT) and peroxidase (POX) patterns were assayed by incubating the native gel with hydrogen peroxide (H_2_O_2_) that was used as substrate, then stained with Potassium Iodide (KI) (Siciliano and Shaw 1976) for detecting the CAT types that appeared as yellow bands and stained by Benzidine (Rescigno et al. 1997) for detecting the POX types that appeared as brown bands. The electrophoretic esterase (EST) patterns were assayed by incubating the native gel in conditioning buffer for optimizing the enzyme activity, then stained with a reaction mixture containing Fast Blue RR (as a dye coupler) along with α- and β-naphthyl acetate (as substrates) for detecting α- and β-EST types, respectively (Ahmad et al. 2012). The α-EST types appeared as brown bands and the β-EST types as pink bands.

#### Data analysis

After photographing the PAGE plates, the Quantity One software (Version 4.6.2) was used for analyzing the relative mobility (Rf), intensity (Int.), percent (B%) and quantity (Qty) of the electrophoretically separated bands. The equation suggested by Nei and Li (1979) was used for calculating percentages of the similarity index (SI%) and genetic distance (GD%).

### Statistical analysis

A one-way analysis of variance test (one-way ANOVA) was used to assess the obtained data statistically. The differences were considered statistically significant when the “*P*” values between the groups were less than 0.05. The results are presented in Tables and Figures as mean ± standard error (SE).

## Results

### The major phyto-constituents and the in vitro biological activities of ethanolic *T. chebula* extract

It was noticed that polyphenolic compounds and condensed tannins are the most common phyto-constituents in the ethanolic *T. chebula* fruit extract. As a result, these constituents were quantified. As depicted in Table 1, it was found that *T. chebula* fruit extract contains high concentrations of total polyphenols (87.82 ± 0.02 mg gallic acid/100 gm) and total condensed tannins (32.52 ± 0.01 μg/mL). The in vitro biological activities of the ethanolic *T. chebula* extract were measured by assaying TAC and IRP. It was found that the extract possessed high TAC (222.82 ± 0.02 mg gallic acid/gm) and IRP (92.84 ± 0.01 µg/mL). Furthermore, the scavenging activity against DPPH and ABTS radicals was determined and compared to that of ascorbic acid (standard). The scavenging activity against DPPH radicals was expressed as IC_50_ values. The low IC_50_ value indicated strong antioxidant activity. The IC_50_ value of the ethanolic *T. chebula* extract was 4.98 ± 0.07 μg/mL and the IC_50_ of the standard ascorbic acid was 3.77 ± 0.01 μg/mL. Moreover, it possessed high scavenging activity against the ABTS radical (31.98 ± 0.07%) as compared to the efficiency of standard ascorbic acid (38.87 ± 0.01%).

**Table 1:** Concentrations of the major active phyto-constituents and the in vitro antioxidant and radical scavenging activities of the ethanolic *Terminalia chebula* fruit extract

	Major Phyto-constituents		Antioxidant activity		Scavenging activity	
	Total polyphenols (mg gallic acid/100 gm)	Total condensed tannins (μg/mL)	TAC (mg gallic acid/gm)	IRP (µg/mL)	DPPH (IC_50_ µg/mL)	ABTS (%)
Extract	87.82 ± 0.02	32.52 ± 0.01	222.82 ± 0.02	92.84 ± 0.01	4.98 ± 0.07	31.98 ± 0.07
STD	–	–	–	–	Ascorbic acid	
					3.77 ± 0.01	38.87 ± 0.01

The anti-diabetic activity of the* T. chebula* extract was determined by assaying the inhibitory effect against α-amylase and α-glucosidase enzymes and comparing it to efficiency of acarbose, which used as a standard drug. In our study, *T. chebula* extract exhibited a moderate inhibitory effect on α-amylase (39.85 ± 0.15%) and α-glucosidase activity (29.59 ± 0.02%) and was compared to the efficiency of acarbose against α-amylase (65.99 ± 0.04%) and α-glucosidase activity (51.19 ± 0.01%) at equal concentrations to *T. chebula* extract. Regarding the anti-Alzheimer activity, the* T. chebula* extract exhibited high inhibitory effect on AChE activity by 47.19 ± 0.01% compared to the efficiency of donepezil (67.28 ± 0.61%) that is used as a standard drug. The anti-inflammatory activity of the* T. chebula* extract was assessed by determining the percentage of protein denaturation and proteinase inhibition. The plant extract showed high inhibition percent (%) against protein denaturation (35.14 ± 0.02%) and proteinase activity (32.88 ± 0.02%) compared to diclofenac sodium (a non-steroidal anti-inflammatory standard drug), which exhibited an inhibitory effect on protein denaturation (42.93 ± 0.01%) and proteinase activity (40.78 ± 0.03%) (Table 2).

**Table 2:** The in vitro anti-diabetic, anti-Alzheimer and anti-inflammatory activities of the ethanolic *Terminalia chebula* fruit extract

	Inhibition (%)			Anti-inflammatory activity	
	Anti-diabetic activity		Anti-Alzheimer activity		
	α-amylase	α-Glucosidase	AChE	Inhibition (%) of Proteinase denaturation	Inhibition (%) of proteinase enzyme
Extract	39.85 ± 0.15	29.59 ± 0.02	47.19 ± 0.01	35.14 ± 0.02	32.88 ± 0.02
STD	Acarbose		Donepezil	Diclofenac sodium	
	65.99 ± 0.04	51.19 ± 0.01	67.28 ± 0.61	42.93 ± 0.01	40.78 ± 0.03

Values were calculated from three replicates (mean ± SE)

### Biochemical assays

During the current study, it was found that markers of oxidative stress were determined by quantifying levels of antioxidants (TAC and GSH) and products of the peroxidation reactions (LPO and TPC) in the brain tissue homogenates (Table 3). It was noticed that there was a decline in levels of TAC and GSH with elevating levels of LPO and TPC significantly (*P* ≤ 0.05) in the brain of the diabetic group compared to the control group. The present study clarified the reduction in oxidative stress induced by STZ via ethanolic *T. chebula* extract at both doses, as evidenced by elevating levels of the antioxidants and decreasing the peroxidation products. The *T. chebula* extract showed a higher ameliorative effect when administered at a dose of 600 mg/kg compared to the metformin that was used as the standard drug.

**Table 3:** Effect of ethanolic* Terminalia chebula* fruit extract against STZ-induced diabetes on markers of oxidative stress in brain tissue of rats

	C	Diabetic	Diabetic treated with		
			Metformin (150 mg/kg)	*T. chebula* (400 mg/kg)	*T. chebula* (600 mg/kg)
*Antioxidants*					
TAC (µmol/g)	40.82 ± 0.08	7.63 ± 0.01^a^	37.55 ± 0.07^ab^	21.22 ± 0.04^abc^	37.14 ± 0.07^ab^
GSH (mg/g tissue)	92.08 ± 2.17	29.43 ± 1.21^a^	78.95 ± 0.80^ab^	66.20 ± 1.45^abc^	84.53 ± 0.80^ab^
*Peroxidation products*					
LPO (nmol/g)	25.75 ± 0.78	158.88 ± 2.50^a^	68.28 ± 1.69^ab^	83.53 ± 2.48^abc^	63.55 ± 1.08^ab^
TPC (nmol/mg protein)	22.00 ± 0.04	117.66 ± 0.24^a^	23.92 ± 0.05^ab^	56.94 ± 0.11^abc^	24.18 ± 0.05^ab^

Data were calculated from five replicates (mean ± SE), a: significant difference from the control group, b: significant difference from the diabetic group, and c: significant difference from the diabetic group treated with metformin (at *P* ≤ 0.05)

Levels of inflammatory markers (IL-1β and TNF-α), β-amyloid and Aβ fragment contents, MCP-1 and activity of the ACHE enzyme that are strongly related to the integrity of brain tissue were elevated significantly (*P* ≤ 0.05) in that tissue of the diabetic rats compared to the control group (Table 4). Administration of *T. chebula* extract improved brain integrity by lowering levels of these measurements in a dose-dependent manner. The extract showed a higher ameliorative effect when administered at a dose of 600 mg/kg compared to metformin. Although the *T. chebula* extract at both doses decreased the levels of these measurements, it could not restore their levels to normal values.

**Table 4:** Effect of ethanolic* Terminalia chebula* fruit extract against STZ-induced diabetes on inflammatory markers (interleukin-1β and tumor necrosis factor-α), β-amyloid (Aβ) contents and activity of the acetylcholineesterase (ACHE) enzyme in brain tissue of rats

	C	Diabetic	Diabetic treated with		
			Metformin (150 mg/kg)	*T. chebula* (400 mg/kg)	*T. chebula* (600 mg/kg)
IL-1β (Pg/g)	74.28 ± 0.86	187.30 ± 1.77^a^	89.60 ± 1.54^ab^	111.13 ± 1.65^abc^	84.55 ± 0.76^ab^
TNF-α (Pg/g)	14.28 ± 0.87	76.30 ± 0.73^a^	23.90 ± 0.40^ab^	38.00 ± 1.12^abc^	27.13 ± 1.00^ab^
Aβ (Pg/g)	8.06 ± 0.04	24.88 ± 0.14^a^	9.65 ± 0.05^ab^	16.68 ± 0.09^abc^	10.31 ± 0.06^abc^
Aβ fragment 1–42 (Pg/g)	34.70 ± 1.00	126.08 ± 1.16^a^	52.05 ± 1.03^ab^	72.95 ± 0.82^abc^	44.15 ± 0.65^abc^
MCP-1 (Pg/g)	115.08 ± 0.93	256.53 ± 4.21^a^	157.35 ± 1.79^ab^	186.30 ± 1.40^abc^	149.33 ± 2.00^ab^
ACHE (ng/g)	59.25 ± 1.31	144.95 ± 2.24^a^	75.25 ± 1.06a^b^	98.80 ± 2.26^abc^	73.30 ± 1.59^ab^

Data were calculated from five replicates (mean ± SE), a: significant difference from the control group, b: significant difference from the diabetic group, and c: significant difference from diabetic group treated with metformin (at *P* ≤ 0.05)

### Histopathological examination

**Fig. 1:**
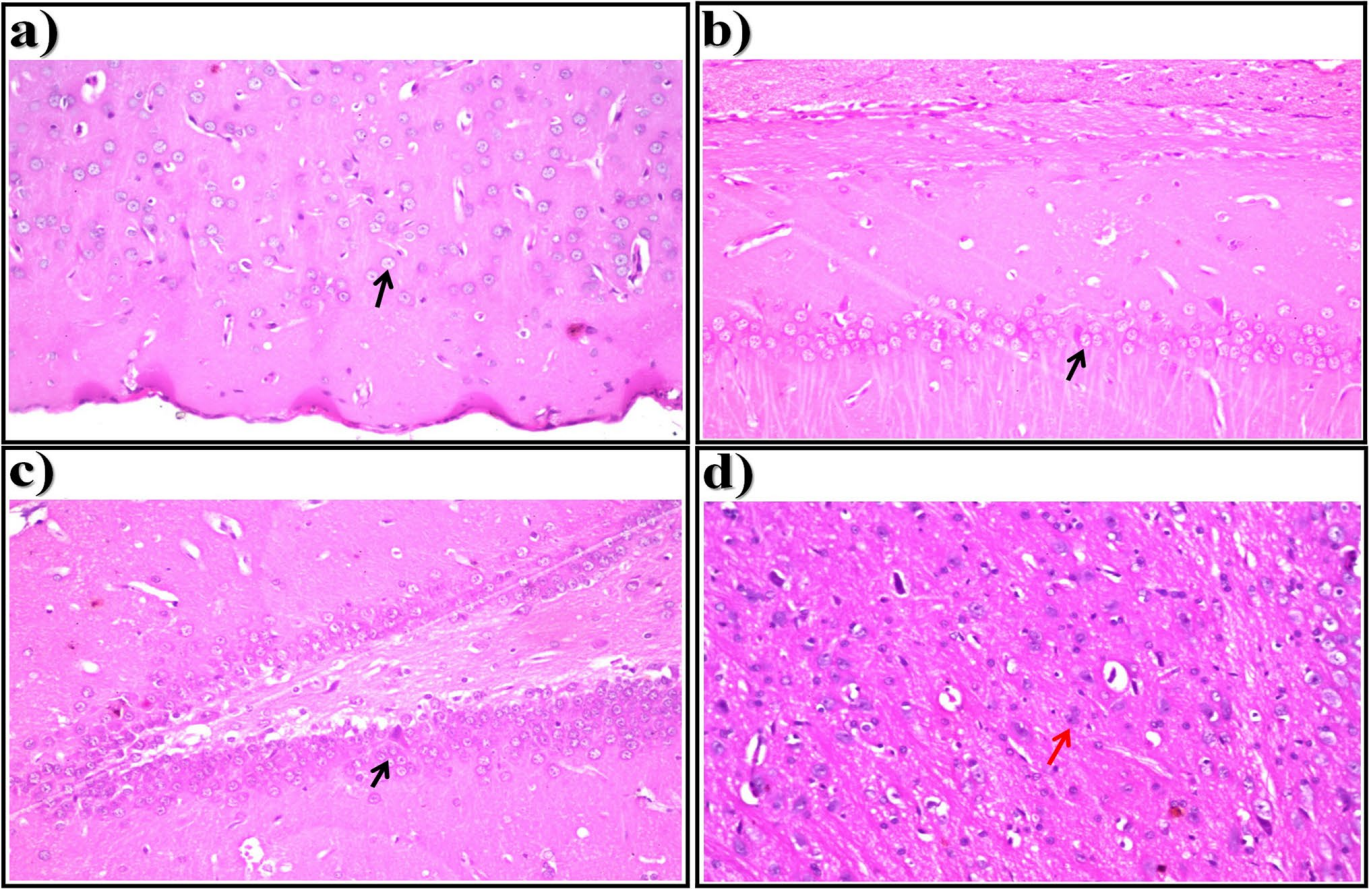
Representative photomicrographs showing. **a** Cerebral Cortex, **b** Subiculum in Hippocampus,** c** Fascia Dentata and Hilus in Hippocampus, and **d** Striatum in brain tissue (H &E-stained) of control rats

**Fig. 2:**
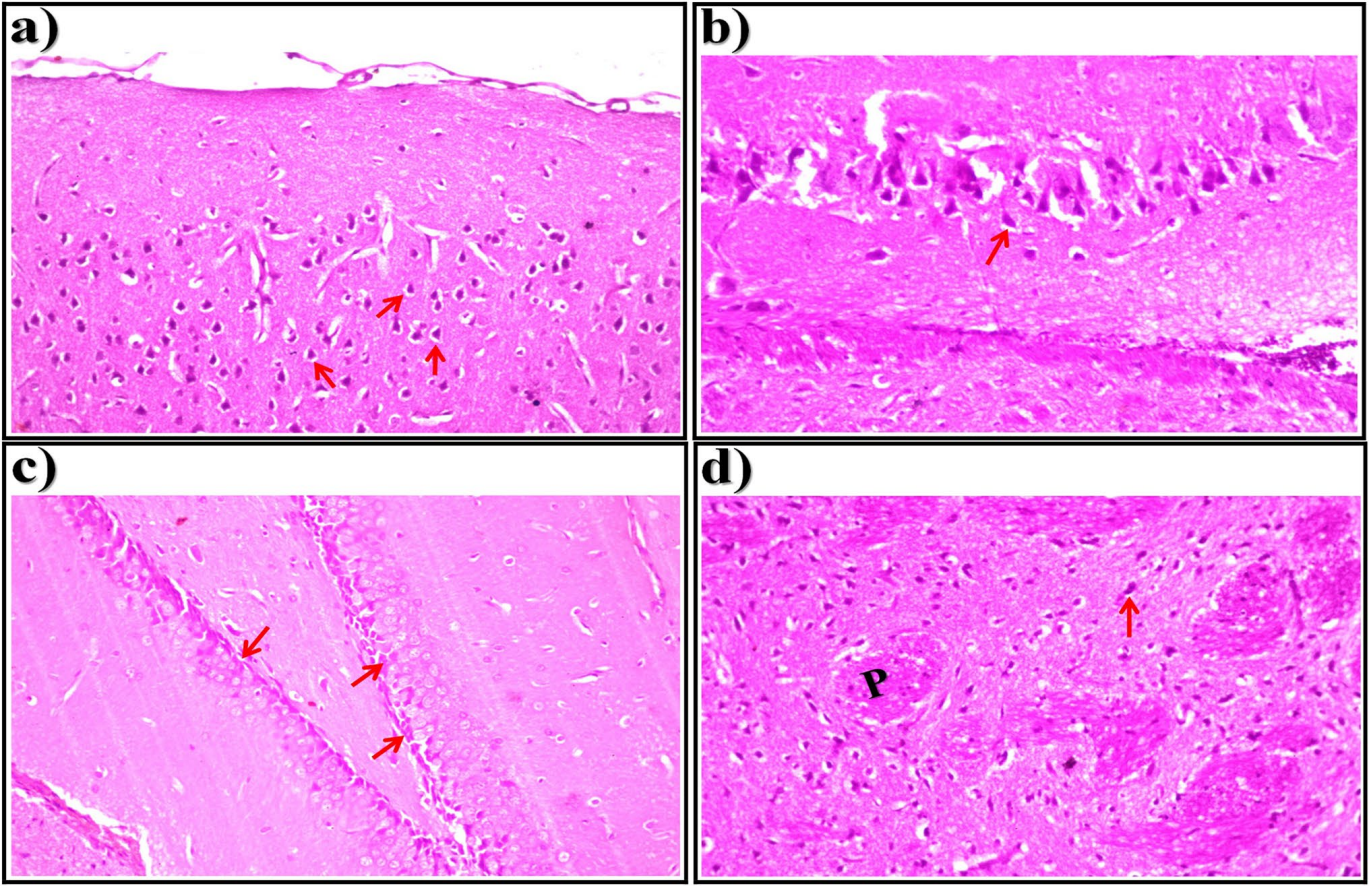
Representative photomicrographs showing. **a** Cerebral Cortex, **b** Subiculum in Hippocampus,** c** Fascia Dentata and Hilus in Hippocampus, and **d** Striatum in brain tissue (H &E-stained) of STZ-induced diabetic rats

Light microscopic examination of brain sections of control rats showed normal histological structure of the neurons (arrows) in the cerebral cortex (Fig. 1a), subiculum in the hippocampus (Fig. 1b), fascia dentata and hilus in the hippocampus (Fig. 1c), and striatum in brain tissue (Fig. 1d). In brain sections of STZ-induced diabetic rats, showed nuclear pyknosis and degeneration (arrows) were seen in all of the neurons in the cerebral cortex (Fig. 2a), in most of the neurons in the subiculum in hippocampus (Fig. 2b), and in a few neurons in the fascia dentata and hilus in the hippocampus (Fig. 2c). As illustrated in Fig. 2d, nuclear pyknosis and degeneration (arrows) were detected in most of the neurons associated with eosinophilic plague (P) formation in multiple focal forms in the striatum of brain tissue.

**Fig. 3:**
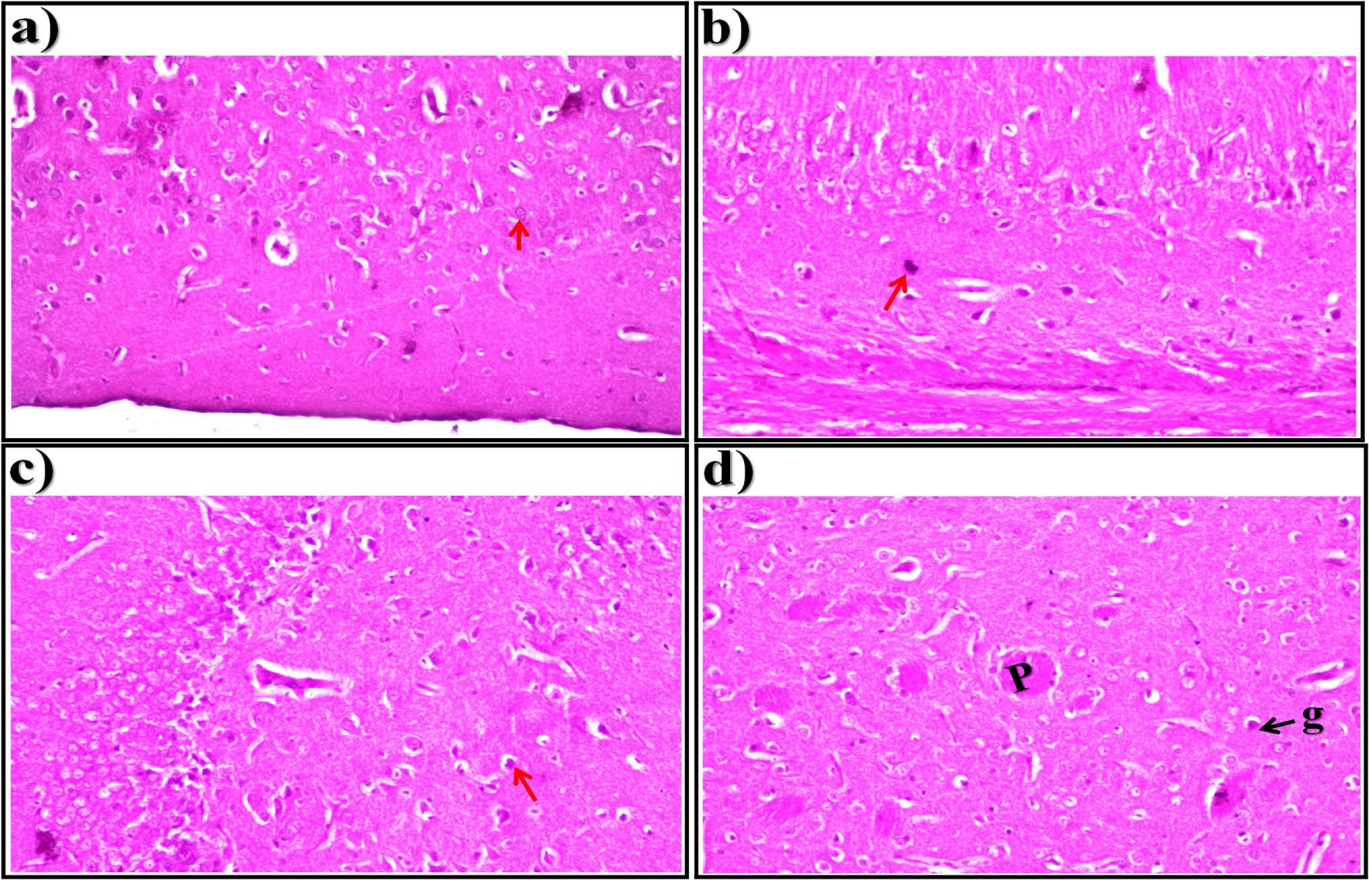
Representative photomicrographs showing. **a** Cerebral Cortex, **b** Subiculum in Hippocampus,** c** Fascia Dentata and Hilus in Hippocampus, and **d** Striatum in brain tissue (H &E-stained) of STZ-induced diabetic rats treated with metformin (standard drug)

In brain sections of STZ-induced diabetic rats treated with metformin, the histological structure of the neurons (arrows) in the cerebral cortex (Fig. 3a), nuclear pyknosis and degeneration (arrows) in a few neurons in the subiculum in the hippocampus (Fig. 3b), and fascia dentata and hilus in the hippocampus (Fig. 3c) were normal. In the striatum of brain tissue, a few eosinophilic plagues (P) formation were detected associated with glia cell proliferation (g) in between (Fig. 3d).

**Fig. 4:**
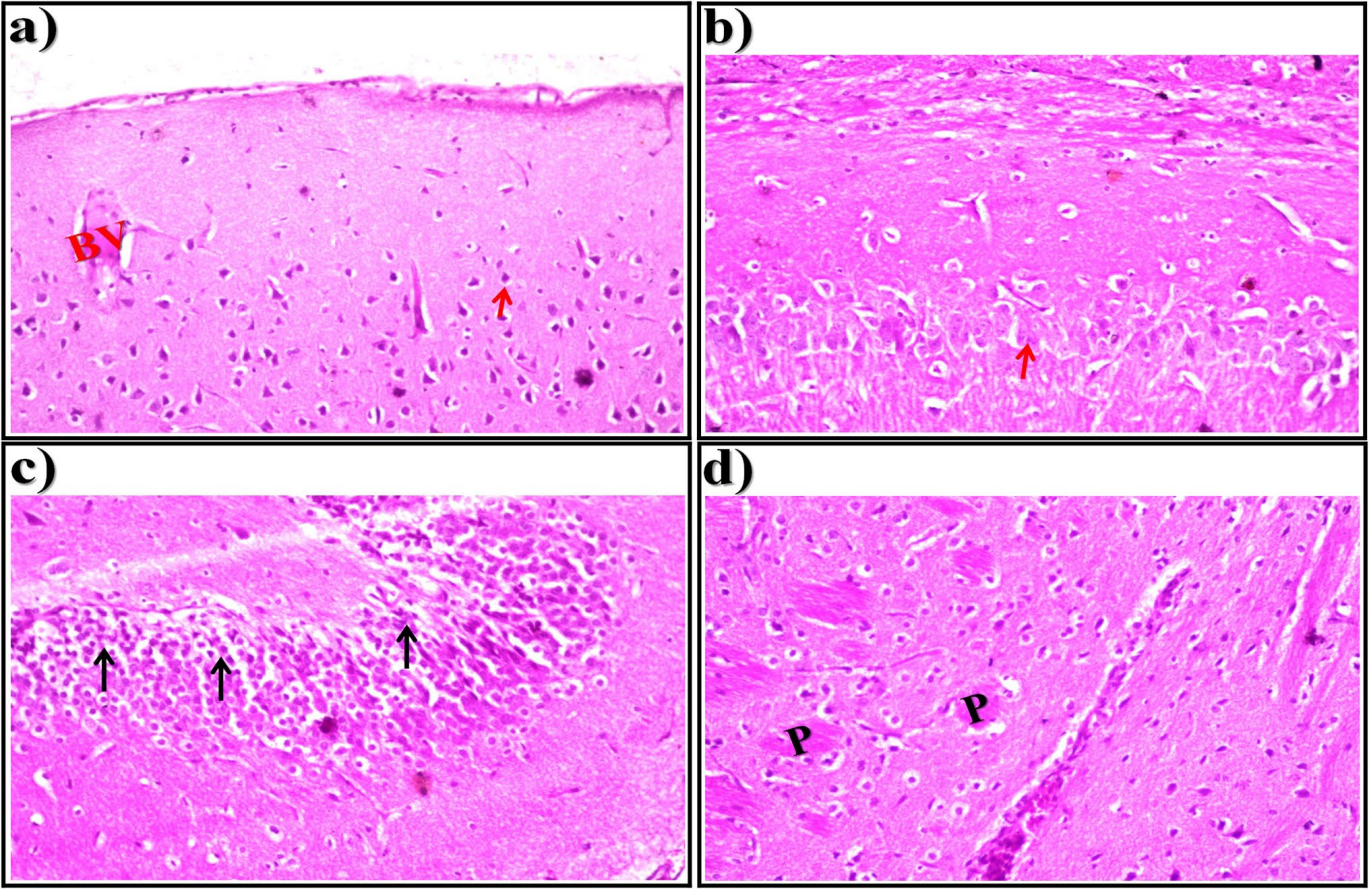
Representative photomicrographs showing. **a** Cerebral Cortex, **b** Subiculum in Hippocampus,** c** Fascia Dentata and Hilus in Hippocampus, and **d** Striatum in brain tissue (H &E-stained) of STZ-induced diabetic rats treated with ethanolic *T. chebula* extract at a dose of 400 mg/kg

In brain sections of STZ-induced diabetic rats treated with ethanolic *T. chebula* extract at a dose of 400 mg/kg, nuclear pyknosis and degeneration (arrows) were seen in most of the neurons in association with congestion in the blood vessels (BV) in the cerebral cortex (Fig. 4a), normal histological structure of the neurons in the subiculum in the hippocampus (Fig. 4b), and nuclear pyknosis and degeneration (arrows) in some neurons in the fascia dentata and hilus in the hippocampus (Fig. 4c). In the striatum of brain tissue, eosinophilic plagues (P) formation was detected with gliosis (Fig. 4d).

**Fig. 5:**
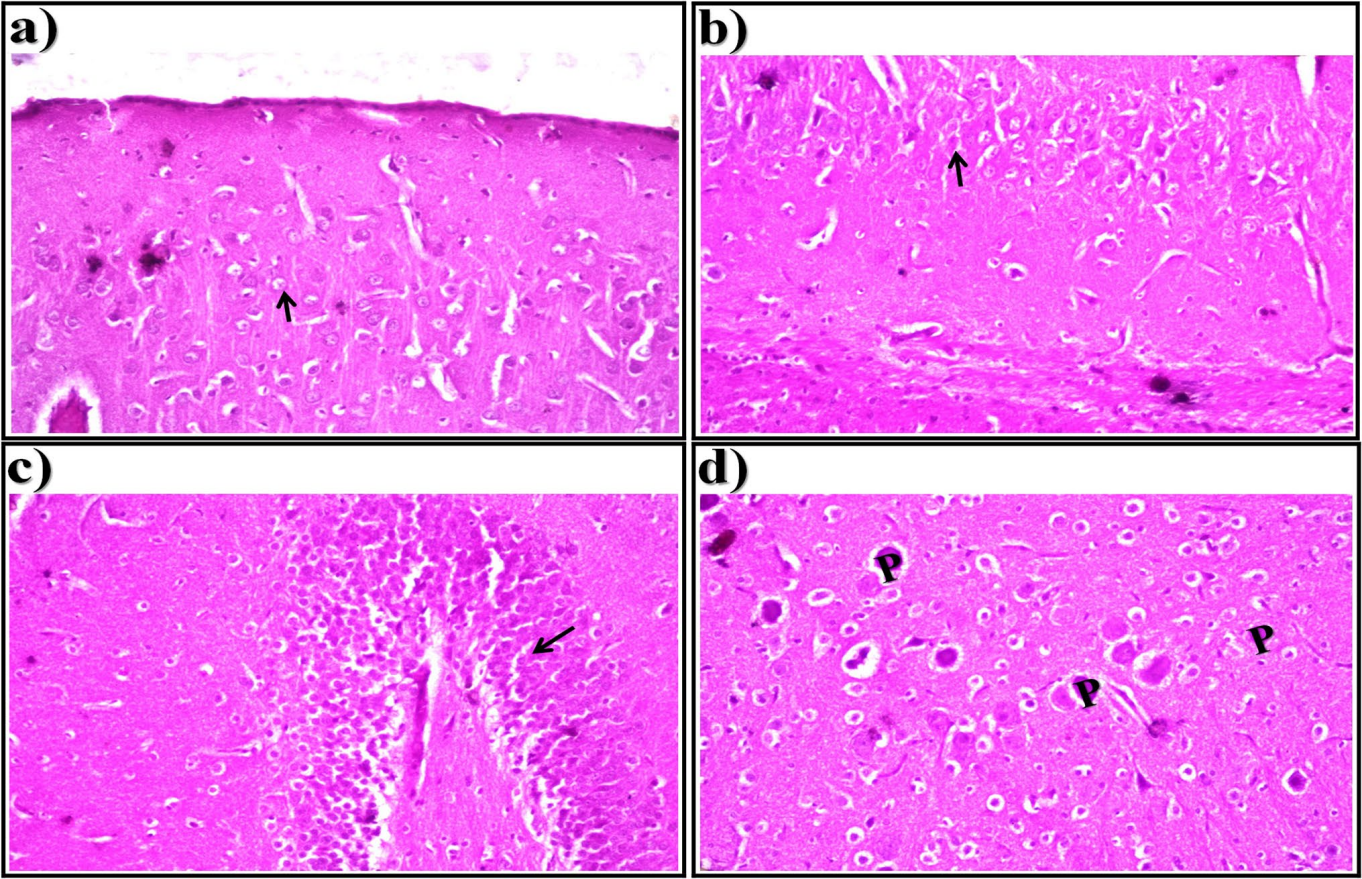
Representative photomicrographs showing. **a** Cerebral Cortex, **b** Subiculum in Hippocampus,** c** Fascia Dentata and Hilus in Hippocampus, and **d** Striatum in brain tissue (H&E-stained) of STZ-induced diabetic rats treated with ethanolic *T. chebula* extract at a dose of 600 mg/kg

In brain sections of STZ-induced diabetic rats treated with the extract at a dose of 600 mg/kg, the histological structure of the neurons (arrows) in the cerebral cortex (Fig. 5a), subiculum in the hippocampus (Fig. 5b), and fascia dentata and hilus in the hippocampus (Fig. 5c) was normal. As presented in Fig. 5d, multiple focal plagues (P) formation was detected in the striatum of brain tissue.

Data presented in Table 5 summarizes scores of the histopathological lesions in brain tissue in rats of different experimental groups, and it was found that the alterations represented by the nuclear pyknosis and degeneration that occurred severely in the cerebral cortex, hippocampus (subiculum, fascia dentata and hilus) and striatum in brain tissue. The extract exhibited the highest ameliorative effect by decreasing the severity of the alterations when administered at a dose of 600 mg/kg compared to the other treated groups.

**Table 5:** Histopathological score showing the effect of ethanolic* Terminalia chebula* fruit extract against the lesions caused by STZ-induced diabetes in the brain tissue of rats

Histopathological lesion			C	Diabetic	Diabetic treated with		
					Metformin (150 mg/kg)	*T. chebula* (400 mg/kg)	*T. chebula* (600 mg/kg)
Nuclear Pyknosis and degeneration of the neurons	Cerebral cortex		−	+++	−	+ +	−
	Hippocampus	Subiculum	−	+ +	+	−	−
		Fascia Dentata	−	+++	+	+	−
	Striatum		−	+++	+ +	+	+ +

−: Nil (0–25%), +: Mild (25–50%), ++: Moderate (50–75%),  +++: Severe (75–100%)

### Electrophoretic assays

#### Electrophoretic protein pattern

**Fig. 6:**
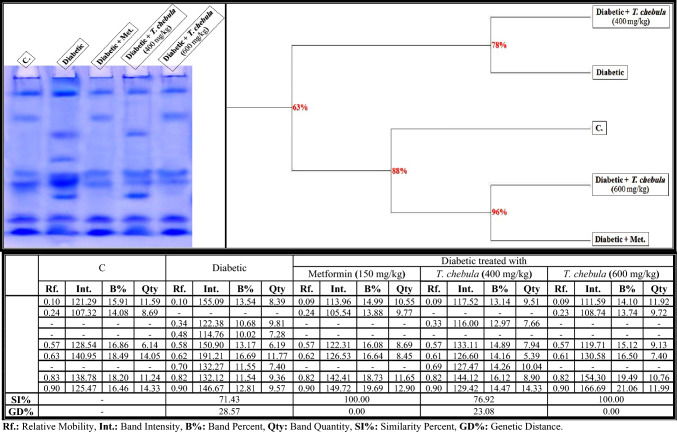
Native electrophoretic protein pattern showing the physiological effect of ethanolic* Terminalia chebula* fruit extract against STZ-induced diabetes on the number and arrangement of the enzyme bands in the brain tissue of rats. Rf.: Relative Mobility, Int.: Band Intensity, B%: Band Percent, Qty: Band Quantity, SI%: Similarity Percent, GD%: Genetic Distance Percent

As shown in Fig. 6, the native protein pattern was represented electrophoretically in the brains of control rats by six bands (Rfs 0.10, 0.24, 0.57, 0.63, 0.83, and 0.90; Int. 121.29, 107.32, 128.54, 140.95, 138.78, and 125.47; B% 15.91, 14.08, 16.86, 18.49, 18.20, and 16.46; Qty 11.59, 8.69, 6.14, 14.05, 11.24, and 14.33, respectively). The five bands that were identified at Rfs 0.10, 0.57, 0.63, 0.83, and 0.90 are considered common bands. In the diabetic group, the physiological alterations in the protein pattern were represented by hiding one normal protein band with the appearance of three abnormal ones (Rfs 0.34, 0.48, and 0.70; Int. 122.38, 114.76, and 132.27; B% 10.68, 10.02, and 11.55; Qty 9.81, 7.28, and 7.40, respectively). The second abnormal band (identified at Rf 0.48) is considered characteristic band. Therefore, SI values (SI = 71.43%; GD = 28.57%) decreased in the brain of the diabetic group compared to the control group.

Administration of the ethanolic* T. chebula* extract (400 mg/kg) minimized the abnormalities induced in the brains of diabetic rats by hiding only the characteristic band without restoring the absent (normal) band. The other abnormal bands still exist at Rfs 0.33 and 0.69 (Int.116.00 and 127.47; B% 12.97 and 14.26; Qty 7.66 and 10.04, respectively). The SI value increased slightly in that group (SI = 76.92%; GD = 23.08%) compared to the diabetic group. The extract at a dose of 600 mg/kg ameliorated the protein pattern by restoring the absent (normal) band (Rf 0.23; Int. 108.74; B% 13.74; Qty 9.72) with hiding the three abnormal ones. Therefore, this group became physiologically similar to the control group (SI = 100.00%; GD = 0.00%).

As presented in Fig. 13a, it was found that there were quantitative changes in the native electrophoretic protein pattern of the diabetic group, where quantities of total bands increased significantly (*P* < 0.05) in that group. The ethanolic* T. chebula* extract (at a dose of 400 mg/kg) minimized the qualitative alterations compared to the control group. It prevented the qualitative abnormalities completely and restored the quantity of total bands to normalcy when administered at a dose of 600 mg/kg.

#### Electrophoretic lipid moiety of native protein pattern

**Fig. 7:**
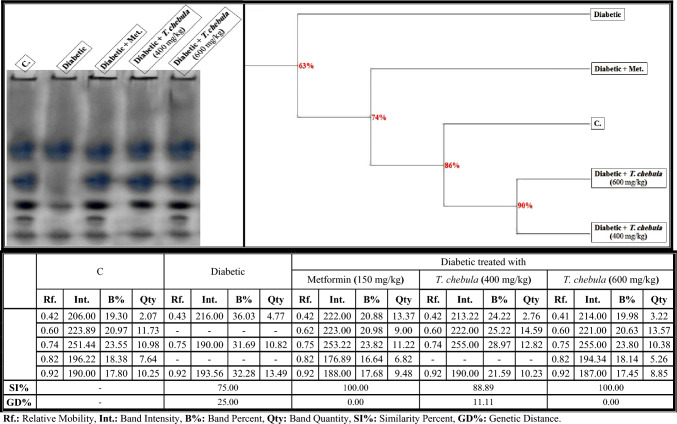
Electrophoretic lipid moiety of native protein pattern showing the physiological effect of ethanolic* Terminalia chebula* fruit extract against STZ-induced diabetes on the number and arrangement of the enzyme bands in the brain tissue of rats. Rf.: Relative Mobility, Int.: Band Intensity, B%: Band Percent, Qty: Band Quantity, SI%: Similarity Percent, GD%: Genetic Distance Percent

As revealed in Fig. 7, the lipid moiety of the native protein pattern was represented in the brain of control rats by 5 bands (Rfs 0.42, 0.60, 0.74, 0.82, and 0.92; Int. 206.00, 223.89, 251.44, 196.22, and 190.00; B% 19.30, 20.97, 23.55, 18.38, and 17.80; Qty 2.07, 11.73, 10.98, 7.64 and 10.25, respectively). The three bands that were identified at Rfs 0.42, 0.74, and 0.92 are considered common bands. No characteristic bands were noticed. In the brains of the diabetic group, it was found that the physiological alterations in this native pattern were represented only by hiding two normal bands. Therefore, SI values (SI = 75.00%; GD = 25.00%) decreased in that group compared to the control group.

Administration of the *T. chebula* extract (400 mg/kg) minimized the abnormalities induced in the brains of diabetic rats by restoring only one band of the two absent (normal) ones identified at Rf 0.60 (Int. 222.00; B% 25.22; Qty 14.59). The SI value increased slightly in that group (SI = 88.89%; GD = 11.11) compared to the diabetic group. Regarding the *T. chebula* extract at a dose of 600 mg/kg, it was noticed that it improved this native protein pattern by restoring the absent (normal) bands identified at Rfs 0.60 and 0.82; Int. 221.00 and 194.34; B% 20.63 and 18.14; Qty 13.57 and 5.26, respectively). Therefore, this group was completely similar to the control group (SI = 100.00%; GD = 0.00%) in terms of metformin.

Data illustrated in Fig. 13a showed that there were quantitative differences in the lipid moiety of the native protein pattern of the diabetic group, where quantities of total bands decreased significantly (*P* < 0.05) in that group. Compared to the control group, the *T. chebula* extract (400 mg/kg) minimized the qualitative changes, but it prevented the qualitative abnormalities completely and restored the quantity of total bands to normalcy when administered at a dose of 600 mg/kg.

#### Electrophoretic calcium moiety of native protein pattern

**Fig. 8:**
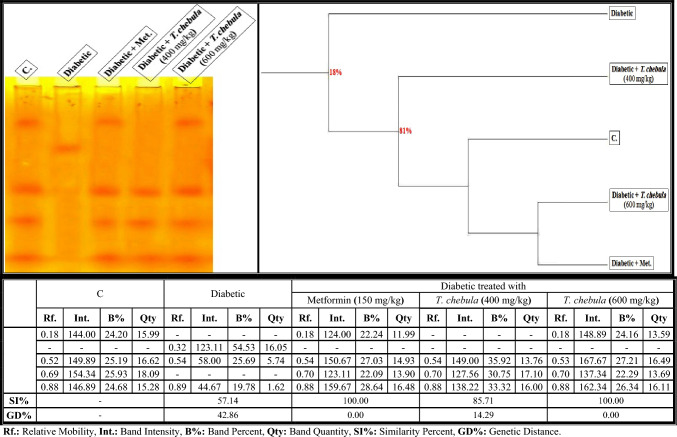
Electrophoretic calcium moiety of native protein pattern showing the physiological effect of ethanolic* Terminalia chebula* fruit extract against STZ-induced diabetes on the number and arrangement of the enzyme bands in the brain tissue of rats. Rf.: Relative Mobility, Int.: Band Intensity, B%: Band Percent, Qty: Band Quantity, SI%: Similarity Percent, GD%: Genetic Distance Percent

As illustrated in Fig. 8, the calcium moiety of the native protein pattern was represented in the brains of control rats by 4 bands (Rfs 0.18, 0.52, 0.69, and 0.88; Int. 144.00, 149.89, 154.34, and 146.89; B% 24.20, 25.19, 25.93, and 24.68; Qty 15.99, 16.62, 18.09, and 15.28, respectively). The normal bands (2) identified at Rfs 0.52 and 0.88 are considered common bands. In the brain of the diabetic group, it was found that the physiological abnormalities in this native pattern were represented by hiding two normal bands with the existence of one characteristic (abnormal) (Rf 0.32; Int. 123.11; B% 54.53; Qty 16.05). Therefore, SI values (SI = 57.14%; GD = 42.86%) decreased in that group compared to the control group.

The *T. chebula* extract (at a dose of 400 mg/kg) minimized the electrophoretic alterations induced in the brain of diabetic rats by hiding the abnormal (characteristic) band and restoring only one band of the two absent (normal) ones identified at Rf 0.70 (Int. 127.56; B% 30.75; Qty 17.10). The SI value increased slightly in that group (SI = 85.71%; GD = 14.29%) compared to the diabetic group. The extract improved this native protein pattern by hiding the abnormal (characteristic) band and restoring the absent (normal) bands (Rfs 0.18 and 0.70; Int. 148.89 and 137.34; B% 24.16 and 22.29; Qty 13.59 and 13.69, respectively) when administered at a dose of 600 mg/kg. Therefore, this group became qualitatively similar to the control group by 100.00% (GD = 0.00%) with metformin.

As presented in Fig. 13a, it was emphasized that the quantitative abnormalities in the calcium moiety of the native protein pattern of the diabetic group were represented by lowering quantities of total bands significantly (*P* < 0.05). The *T. chebula* extract (at a dose of 400 mg/kg) decreased the qualitative alterations, but it prevented the qualitative abnormalities completely and restored the quantity of total bands to normal values when administered at a dose of 600 mg/kg.

#### Electrophoretic catalase (CAT) pattern

**Fig. 9:**
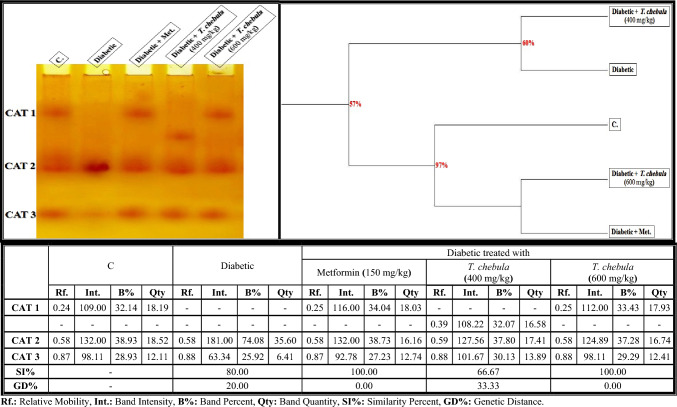
Native electrophoretic Catalase (CAT) isoenzymes pattern showing the physiological effect of ethanolic* Terminalia chebula* fruit extract against STZ-induced diabetes on the number and arrangement of the enzyme bands in the brain tissue of rats. Rf.: Relative Mobility, Int.: Band Intensity, B%: Band Percent, Qty: Band Quantity, SI%: Similarity Percent, GD%: Genetic Distance Percent

As illustrated in Fig. 9, the CAT isoenzyme pattern in the brain of control rats was represented by 3 types (Rfs 0.24, 0.58, and 0.87; Int. 109.00, 132.00, and 98.11; B% 32.14, 38.93, and 28.93; Qty 18.19, 18.52, and 12.11, respectively). The two CAT types (CAT2 and CAT3) that were identified at Rfs 0.58 and 0.87 are considered common bands. Only one characteristic (unique) band was identified in the diabetic group treated with* T. chebula* extract (400 mg/kg) at Rf 0.39 (Int. 108.22; B% 32.07; Qty 16.58). In the brain of the diabetic group, it was found that the qualitative alterations in this isoenzyme pattern were represented by hiding one normal CAT (CAT1) type. Therefore, SI values (SI = 80.00%; GD = 20.00%) decreased in that group compared to the control group.

The electrophoretic alterations in the CAT isoenzyme pattern increased in the diabetic group treated with the *T. chebula* extract at a dose of 400 mg/kg due to the existence of the characteristic band. Therefore, the lowest SI exists in that group (SI = 66.67%; GD = 33.33%) compared to the diabetic group. The *T. chebula* extract improved the CAT isoenzyme pattern by restoring the absent (CAT1) band (Rf 0.25; Int. 112.00; B% 33.43; Qty 17.93) when administered at a dose of 600 mg/kg. Therefore, this group was completely similar to the control group (SI = 100.00%; GD = 0.00%) in terms of metformin.

The quantitative changes in the CAT isoenzyme pattern of the diabetic group were represented by a significant (*P* < 0.05) decrease in the quantity of total bands. The *T. chebula* extract (at a dose of 400 mg/kg) decreased the qualitative alterations by increasing the quantity of total bands significantly (*P* < 0.05) but it restored the quantity of total bands to normalcy when administered at a dose of 600 mg/kg (Fig. 13b).

#### Electrophoretic peroxidase (POX) pattern

**Fig. 10:**
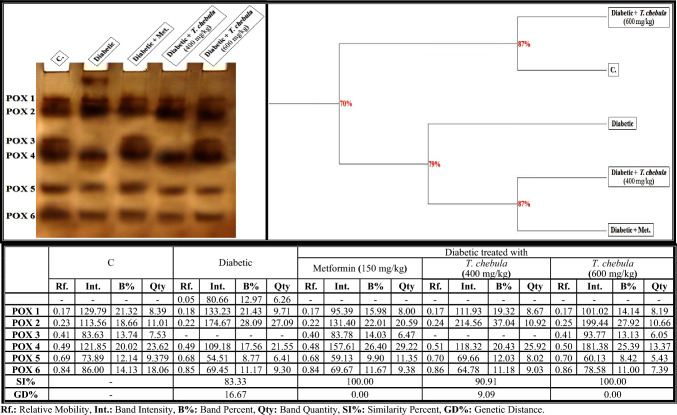
Native electrophoretic Peroxidase (POX) isoenzymes pattern showing the physiological effect of ethanolic* Terminalia chebula* fruit extract against STZ-induced diabetes on the number and arrangement of the enzyme bands in the brain tissue of rats. Rf.: Relative Mobility, Int.: Band Intensity, B%: Band Percent, Qty: Band Quantity, SI%: Similarity Percent, GD%: Genetic Distance Percent

As shown in Fig. 10, the POX isoenzyme pattern in the brain of control rats was represented by six types (Rfs 0.17, 0.23, 0.41, 0.49, 0.69, and 0.84; Int. 129.79, 113.56, 83.63, 121.85, 73.89, and 86.00; B% 21.32, 18.66, 13.74, 20.02, 12.14, and 14.13; Qty 8.39, 11.01, 7.53, 23.62, 9.379, and 18.06, respectively). The POX types (POX1, POX2, POX4, POX5 and POX6) that were identified at Rfs 0.17, 0.23, 0.49, 0.69 and 0.84 are considered common bands. There was only one unique characteristic identified in the diabetic group (Rf 0.05; Int. 80.66; B% 12.97; Qty 6.26). In the brain of the diabetic group, it was found that the qualitative alterations in this isoenzyme pattern were represented by hiding one normal POX (POX3) type with the existence of the characteristic band. Therefore, SI values (SI = 83.33%; GD = 16.67%) decreased in that group compared to the control group.

Administration of the *T. chebula* extract (400 mg/kg) minimized the electrophoretic alterations induced in the brains of diabetic rats by hiding the abnormal (characteristic) band without restoring the absent POX (normal) type. Therefore, the SI value increased slightly in that group (SI = 90.91%; GD = 9.09%) compared to the diabetic group. The *T. chebula* extract ameliorated the POX isoenzyme pattern completely by hiding the abnormal (characteristic) band and restoring the absent (POX3) band (Rf 0.41; Int. 93.77; B% 13.13; Qty 6.05) when administered at a dose of 600 mg/kg. Therefore, this group became physiologically identical to the control group (SI = 100.00%; GD = 0.00%) on metformin.

As illustrated in Fig. 13b, it was noticed that there were quantitative alterations represented in the POX isoenzyme pattern of the diabetic group by elevating the quantity of total bands significantly (*P* < 0.05). The *T. chebula* extract (at a dose of 400 mg/kg) caused a significant (*P* < 0.05) decline in the quantity of total bands, which was restored to normal values when administered at a dose of 600 mg/kg.

#### Electrophoretic esterase (EST) pattern

**Fig. 11:**
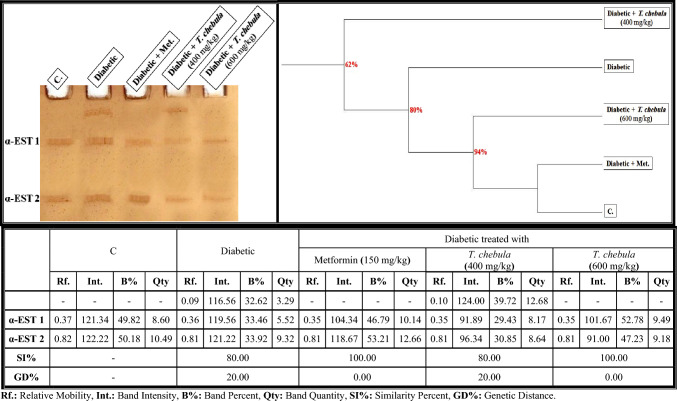
Native electrophoretic α-esterase (α-EST) isoenzymes pattern showing the physiological effect of ethanolic* Terminalia chebula* fruit extract against STZ-induced diabetes on the number and arrangement of the enzyme bands in the brain tissue of rats. Rf.: Relative Mobility, Int.: Band Intensity, B%: Band Percent, Qty: Band Quantity, SI%: Similarity Percent, GD%: Genetic Distance Percent

In the brains of control rats (Fig. 11), the electrophoretic α-EST isoenzyme pattern was represented by 2 types (Rfs 0.37 and 0.82; Int. 121.34 and 122.22; B% 49.82 and 50.18; Qty 8.60 and 10.49, respectively). The two identified normal α-EST types (α-EST1 and α-EST2) are considered common bands. In the brain of the diabetic group, it was found that the alterations occurred qualitatively in this isoenzyme pattern and were represented by an existing abnormal band (Rf 0.09; Int. 116.56; B% 32.62; Qty 3.29). Therefore, SI values (SI = 80.00%; GD = 20.00%) decreased in that group compared to the control group.

Administration of *T. chebula* extract (400 mg/kg) couldn't prevent the physiological abnormalities. Therefore, the SI value in this group (SI = 80.00%; GD = 20.00%) was equal to that in the diabetic group compared to the control group. The extract ameliorated this isoenzyme pattern completely by hiding the abnormal band when administered at a dose of 600 mg/kg. Therefore, this group became physiologically similar to the control group by 100.00% (GD = 0.00%) with metformin.

As revealed in Fig. 13b, it was observed that the alterations occurred quantitatively in the α-EST isoenzyme pattern of the diabetic group by elevating the quantity of total bands significantly (*P* < 0.05). The treatment with *T. chebula* extract at both studied doses (400 and 600 mg/kg) decreased the quantity of total bands significantly (*P* < 0.05) in a dose-dependent manner. The extract restored the quantity of total bands to normalcy only when administered at a dose of 600 mg/kg.

**Fig. 12:**
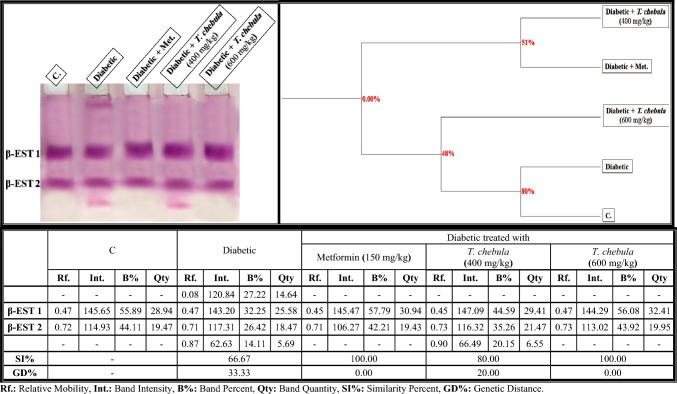
Native electrophoretic β-esterase (β-EST) isoenzymes pattern showing the physiological effect of ethanolic* Terminalia chebula* fruit extract against STZ-induced diabetes on the number and arrangement of the enzyme bands in the brain tissue of rats. Rf.: Relative Mobility, Int.: Band Intensity, B%: Band Percent, Qty: Band Quantity, SI%: Similarity Percent, GD%: Genetic Distance Percent

As shown in Fig. 12, the electrophoretic β-EST isoenzyme pattern was represented in the brain of control rats by 2 types (Rfs 0.47 and 0.72; Int. 145.65 and 114.93; B% 55.89 and 44.11; Qty 28.94 and 19.47, respectively). The two identified normal β-EST types (β-EST1 and β-EST2) are considered common bands. Only one characteristic was identified in the diabetic group (Rf 0.08; Int. 120.84; B% 27.22; Qty 14.64). In the brain of the diabetic group, it was found that the qualitative abnormalities in this isoenzyme pattern were represented by existing two abnormal bands (Rfs 0.08 and 0.87; Int. 120.84 and 62.63; B% 27.22 and 14.11; Qty 14.64 and 5.69, respectively). Therefore, the lowest SI value (SI = 66.67%; GD = 33.33%) exists in that group compared to the control group.

The *T. chebula* extract (at a dose of 400 mg/kg) minimized the physiological abnormalities by hiding one of the two abnormal bands (the characteristic band). Therefore, the SI value in this group increased slightly (SI = 80.00%; GD = 20.00%) when compared to the diabetic group. The extract prevented the qualitative deleterious effect on this isoenzyme pattern completely by hiding the two abnormal bands when administered at a dose of 600 mg/kg. Therefore, this group became completely identical to the control group (SI = 100.00%; GD = 0.00%) as metformin.

**Fig. 13:**
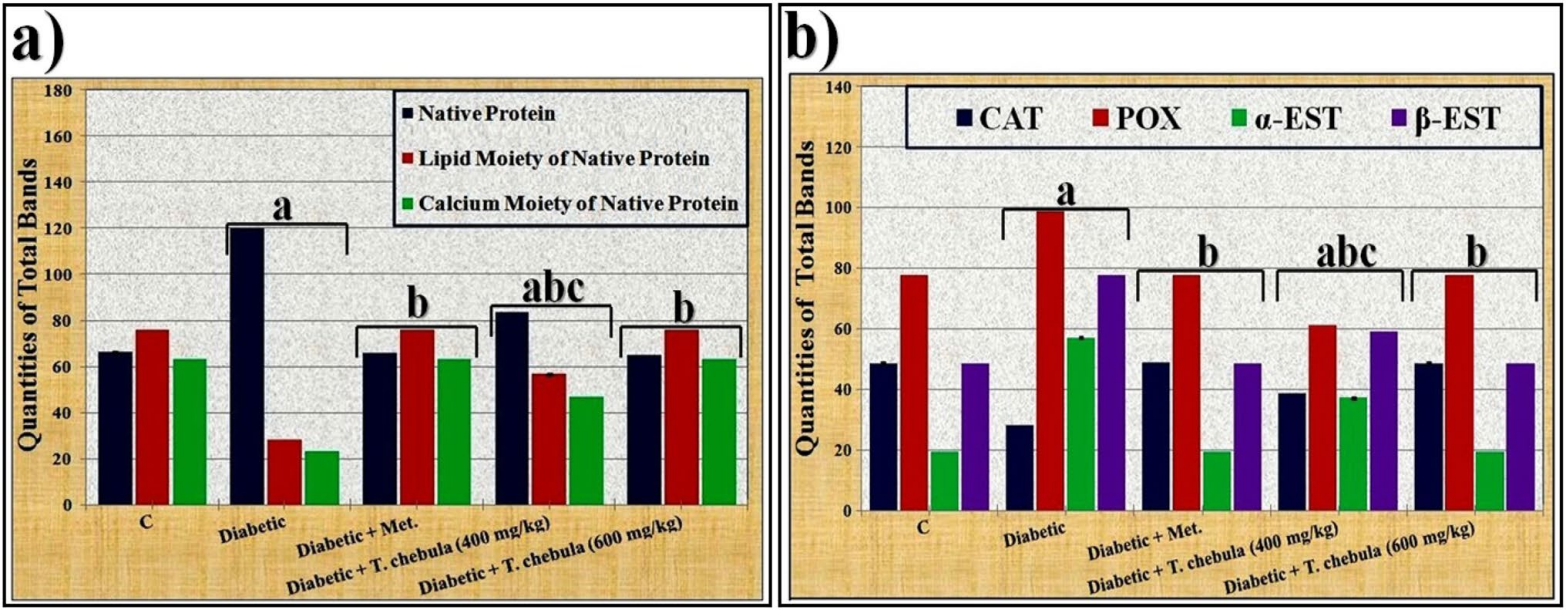
Data showing the ameliorative effect of ethanolic* Terminalia chebula* fruit extract against the quantitative alterations induced by STZ in different electrophoretic **a** Protein, **b** Isoenzymes Patterns in brain tissue of rats. Data were calculated from three replicates (mean ± SE),** a**: significant difference from the control group, **b**: significant difference from the diabetic group, and** c**: significant difference from the diabetic group treated with metformin (at *P* ≤ 0.05)

The alterations occurred quantitatively in the β-EST isoenzyme pattern by elevating the quantity of total bands significantly (*P* < 0.05) in the diabetic group. The treatment with *T. chebula* extract at both doses (400 and 600 mg/kg) decreased the quantity of total bands significantly (*P* < 0.05) in a dose-dependent manner. The quantity of the total bands was restored to normal via administration of the extract at a dose of 600 mg/kg compared to the control group (Fig. 13b).

## Discussion

During the current study, it was noticed that the ethanolic *T. chebula* fruit extract is rich in polyphenolic compounds and total tannins. This agrees with polyphenolic compounds and total tannins. This agreed with Rani et al. (2018) who reported that there is a linear co-relationship between these phenolic compounds and the reducing power of the extract. The antioxidant and iron reducing power of the *T. chebula* extract might be attributed to their phenolic constituents (Saha and Verma 2016). The reduction of DPPH by *T. chebula* extract was either due to the transfer of a hydrogen atom from the phenolic compounds, which are considered effective hydrogen donors (Sheng et al. 2018). The reductive capacity of these phenolic compounds depends on the presence of reductones, which exhibit their potential by breaking the free radical chain and donating a hydrogen atom. Consequently, the radical chain reactions were terminated and may otherwise be very damaging (Sun et al. 2014).

In our study, the *T. chebula* extract possessed a high inhibitory effect on the activities of α-amylase and α-glucosidase compared to acarbose (the standard drug). This agreed with Kifle et al. (2020) and was supported recently by Aboulthana et al. (2022) who demonstrated that the presence of phenolic acids and tannins is responsible for the inhibitory effect on the activities of these enzymes. The native extract exhibited anti-Alzheimer (anti-cholinesterase) activity, and this might be attributed to increasing the antioxidant activities, which are strongly related to the anti-diabetic and anti-Alzheimer activities (Russo et al. 2015). Therefore, the extract that possesses antioxidant activities exhibits anti-diabetic and anti-Alzheimer activities. The anti-inflammatory activity was assayed by measuring the efficiency of the extract in inhibiting protein denaturation and the activity of proteinase enzyme (Hassan et al. 2023). Ability of the extract to inhibit proteinase denaturation and proteinase enzymes refers to the apparent potential for anti-inflammatory activity (Ayman et al. 2023).

The pathogenesis of the brain dysfunction induced as a result of the incidence of diabetes is not fully understood. The brain is the most susceptible organ to glucose fluctuations and inflammation. The hyperglycemia affected both metabolic and vascular pathways, leading to disturbances in widespread brain regions and compromised brain function (Wu et al. 2021). In the early stages of diabetes, cognitive impairment might occur. Therefore, it is necessary to identify key markers of early neuronal dysfunction (Piatkowska-Chmiel et al. 2021).

During the present study, it was noticed that levels of TAC and GSH decreased in the brains of diabetic rats, and this agreed with Tian et al. (2016) who showed that the antioxidants decreased in the brains due to inducing the formation of reactive species via glucose autoxidation and/or glycation of proteins non-enzymatically. Both LPO and TPC were elevated significantly in the brains of diabetic rats due to overproduction of reactive oxygen species (ROS) that interact with lipids and proteins (Pandey and Rizvi 2010). The ethanolic *T. chebula* extract increased the antioxidants and reduced the products of the peroxidation reactions. This was in agreement with Khalaf et al. (2019) who postulated that the extract prevented the alterations induced by oxidative stress and maintained a near normal antioxidant status due to the presence of the active phyto-constituents that can act as singlet oxygen scavengers and hydrogen atom donors. Therefore, they possess antioxidant properties.

Neuroinflammation and oxidative stress are the pathological hallmarks of most neurodegenerative diseases. Activation of astrocytes and microglia as a result of injuries to the central nervous system leads to the subsequent release of proinflammatory cytokines and hence neuronal death (El-Shamarka et al. 2022). The present study revealed that levels of IL-1β, TNF-α and MCP-1 elevated significantly in the brains of diabetic rats and this agreed with Piatkowska-Chmiel et al. (2021) who reported that levels of these pro-inflammatory cytokines increased due to their positive correlation with cognitive disturbances. Mushtaq et al. (2015) added that the production of these cytokines is closely related to accelerating the neurodegeneration process. The pro-inflammatory cytokines were elevated due to the abnormally differentiated vascular endothelia cells and perivascular macrophages, which might reveal an exaggerated inflammatory response characterized by elevating the secretion of these cytokines (Sochocka et al. 2017). Also, DM might be accompanied by exaggerated glial cell activation, which leads to the release of large amounts of inflammatory agents (Khandelwal et al. 2011). The ethanolic *T. chebula* extract ameliorated levels of pro-inflammatory cytokines and this agreed with Jung et al. (2019) who emphasized that the *T. chebula* extract is rich in various chemical constituents like chebulanin, chebulic acid, chebulagic acid, chebulinic acid, corilagin, gallic acid and ellagic acid that exert anti-inflammatory and antioxidant effects in addition to their ability to inhibit histamine secretion.

AChE exhibits its effective role in the cholinergic nervous system by hydrolyzing the neurotransmitter acetylcholine (after completing its role in maintaining memory function) into choline and acetate. Therefore, it is responsible for transporting the nerve signals and terminating synaptic transmission (Contestabile 2011). It was found that levels of ACHE and Aβ contents were elevated significantly in the brains of diabetic group during the current study. This agreed with Ahmed et al. (2011) who emphasized that there is a direct correlation between ACHE and Aβ contents. Therefore, the elevated Aβ binds directly to nicotinic receptors, leading to elevation of the ACHE content in and around Aβ plaques. Moreover, the ACHE is able to form Aβ-ACHE complex (more toxic) after co-localization with Aβ deposits, which consequently promote the assembly of the Aβ into amyloid fibrils (Holmquist et al. 2007). The *T. chebula* extract decreased the activity of ACHE, and this agreed with Mathew et al. (2013) who demonstrated that this fruit was chosen as an efficacious candidate as a source of potent AChE inhibitors as well as antioxidants. Therefore, this plant species is traditionally used for treating Alzheimer’s disease and disorders of the central nervous system. Also, it decreased the Aβ contents due to inhibiting the AChE enzyme, which consequently prevents the formation of β-amyloid plaques (Mathew and Subramanian 2012).

The histopathological examination is used for assessing the brain damage induced as a result of DM incidence, and it was noticed that the hippocampus, which involved in learning and memory, is the most sensitive region to hyperglycemia compared to other brain regions (Zheng et al. 2017). The present study showed that the lesions occurred severely in the cerebral cortex, hippocampus and striatum regions of the brain tissue in diabetic rats. This was in agreement with Huang et al. (2022) who reported that degeneration of the neurons in the thalamic nuclei, cingulate cortex and hippocampus in the brains of diabetic rats might be related to increasing the production of ROS. Yongue et al. (2014) suggested that the changes induced by DM in cognitive function with altering brain activity in the hippocampus region might be attributable to the decreasing number of pyramidal neurons in the rat hippocampus of diabetic rats. The *T. chebula* extract decreased the severity of lesions in brain tissue, and this agreed with Shen et al. (2017) who reported that the plant extract is characterized by the presence of ellagic acid, which is responsible for the neuroprotective efficacy by reducing the influx of calcium ions and inhibiting the production of ROS. Lin et al. (2022) proposed that the total phenolic and tannin content present in *T. chebula* extract exert neuroprotective activity due to their scavenging activities against excessive hydroxyl and peroxyl radicals and improving the antioxidant systems.

The alterations in the protein pattern detected electrophoretically might be related to the oxidative stress and elevated free radical formation induced in diabetic rats by hyperglycemia (Abdel-Halim et al. 2020). It was presumed that malondialdhyde (MDA), a secondary product of lipid peroxidation, altered the protein pattern due to the presence of the aldehyde groups, which act as an anchor between sugar and protein moieties, thereby enhancing the formation of the glycated proteins (Ito et al. 2019). Furthermore, these changes might be attributable to the reaction of the most abundant brain proteins with the ROS, which consequently leads to various chemical changes like fragmentation, oxidation, aggregation and cross-linking of protein molecules (Hawkins et al. 2009). Also, the glycation process reduced the efficiency of the chaperone in exhibiting its biological role by causing protein folding (Adams et al. 2021). The physiological changes in the lipid moiety of native protein pattern in the brains of diabetic rats might refer to overproduction of the ROS that attack the lipid portion, leading to oxidative modifications in the lipid moiety of proteins (El-Sayed et al. 2018). The protein binds naturally to lipoproteins in the brain tissue. Therefore, changing the lipid moiety of the native protein pattern might result in altering the protein pattern in that tissue (Satoh 2005). The calcium moiety of the native protein pattern that is responsible for protecting the tissue and detoxification against toxic agents is altered in the brains of diabetic rats due to the abnormal mineralization in that tissue (Abulyazid et al. 2017). Also, the abnormalities that occurred qualitatively and quantitatively in this protein pattern might be related to the role of the generated ROS in converting an active hydrogen atom from these biomolecules (Abd Elhalim et al. 2017; Aboulthana et al. 2020). The changes in the electrophoretic CAT and POX patterns in the brains of diabetic rats might be attributed to degeneration of the protein contents (Ramanathan et al. 1999), glycation of these enzymes that inhibits their activities (Al-Enazi 2014), and/or due to uncontrolled production of the ROS that affect the protein portion of these enzymes directly and consequently change the physico-chemical properties of the endogenetic CAT and POX enzymes (De Freitas et al. 2014). The brain is rich in ESTs enzymes due to their effective role in the neurotransmission process, where they catalyze the breakdown of acetylcholine liberated during stimulation of the nervous system (Srividhya et al. 2012). Moreover, they have the ability to catalyze the hydrolysis of ester bonds in the neutral lipids introduced into cells as lipid deposits and components of lipoproteins and break them down into the corresponding carboxylic acids (Benjamin et al. 2015). Electrophoresis is the most suitable technique for identifying their molecular forms due to their hydrodynamic properties in addition to the presence of active thiol groups (Abulyazid et al. 2017). In the brains of diabetic rats, the alterations and characteristic changes in the electrophoretic α- and β-EST isoenzyme patterns might be attributable to glycosylation of the EST types that occurred abnormally, leading to protein degradation and hence ESTs instability (Aboulthana et al. 2018). Seif et al. (2017) proposed that the abnormalities in the EST pattern might be attributed to the effect of ROS on the integrity of the protein molecule due to sulfhydral-mediated cross-linking of the labile amino acids, and changing the fractional activity of different isoenzymes seemed to be correlated with the changes in rate of protein expression secondary to DNA damage induced by oxidative stress and overproduction of ROS. No changes occurred in the electrophoretic in α- and β-EST isoenzyme patterns if there were no alterations in the protein expression (Aboulthana et al. 2016; El-Sayed et al. 2018). It has been shown that treatment with *T. chebula* extract ameliorated the physiological abnormalities in all electrophoretic protein (native protein and lipid moiety of native protein) and isoenzyme (CAT, POX, α-Amy, α-EST and β-EST) patterns induced in the brains of diabetic rats due to the presence of phyto-constituents that have the ability to scavenge ROS and could protect these biologically active macromolecules from oxidation (Zhang et al. 2015). Furthermore, the number and location of the hydroxyl groups linked to the phenolic compounds, in addition to the concentration of the phenols, are responsible for stimulating the antioxidant defense against the reactive species targeting these biomacromolecules (Abdel-Halim et al. 2020). In addition, the electrophoretic protein and isoenzymes patterns were restored to their normal state after administering plant extract due to the role of the phyto-constituents that have insulin-like action and exhibit anti-glycating activity through other mechanisms irrespective of glycation inhibition (Akhand et al. 2013).

## Conclusions

The ethanolic *T. chebula* extract is rich in the highest concentrations of total polyphenolic and total tannins, and hence it possesses high antioxidant and scavenging activities. Furthermore, it exhibited high in vitro anti-diabetic, anti-Alzheimer's and anti-inflammatory activities compared to the standard. The extract exhibited the highest ameliorative effect against the biochemical, histopathological and electrophoretic abnormalities induced by STZ in the brains of diabetic rats when administered at a dose of 600 mg/kg compared to the metformin that is used as a standard anti-diabetic drug.

## Data Availability

No Data associated in the manuscript.
